# Challenges of living with veterans with post-traumatic stress disorder from the perspective of spouses: a qualitative content analysis study

**DOI:** 10.1186/s12888-024-05572-y

**Published:** 2024-02-21

**Authors:** Zahra Maddah, Reza Negarandeh, Soheil Rahimi, Shahzad Pashaeypoor

**Affiliations:** 1 Department of Psychiatric Nursing, School of Nursing and Midwifery, Tehran University of Medical Sciences, Tehran, Iran; 2 Nursing & Midwifery Care Research Center, School of Nursing and Midwifery, Tehran University of Medical Sciences, Tehran, Iran; 3 Department of Community Health and Geriatric Nursing, School of Nursing and Midwifery, Tehran University of Medical Sciences, Tehran, Iran; 4 Community Based Participatory Research Center, Tehran University of Medical Sciences, Tehran, Iran

**Keywords:** Post-traumatic stress disorder, Spouses, Veteran, Challenges

## Abstract

**Background and objective:**

The needs and characteristics of veterans with post-traumatic stress disorder (PTSD) create significant challenges in family life, particularly for spouses. Identifying the nature of these challenges from the perspective of spouses leads to a more comprehensive and profound understanding of their existing problems and can be used for targeted interventions. Therefore, this research was conducted to explore the challenges of living with veterans suffering from PTSD from the perspective of their spouses.

**Methods:**

This qualitative study used conventional content analysis to explore Challenges of spouses of veterans with post-traumatic stress disorder. Fifteen spouses of veterans with PTSD from the Veterans Affairs Center in Iran between June 2022 and January 2023, were purposively selected to participate in the study. Semi-structured in-depth interviews were conducted to collect data. The interviews were audio-recorded and transcribed verbatim. The data were analyzed using the method proposed by Graneheim and Lundman content analysis method with the support of MAXQDA 2020 software.

**Results:**

The mean age of the participants was 56.74 ± 6.43 years. Through data analysis, seven main categories and sixteen subcategories were identified. These categories included burnout (sleep disturbances, feelings of exhaustion), apathy towards self-care and caring for the veteran (neglecting self-care, lack of interest in continuing care), depression (feelings of hopelessness and being at the end of the line, decreased self-confidence ( Crushed and ignored (being mistreated, having multiple roles), relationship disturbances (Dissatisfaction with marital relationship,isolation and limited social interactions, disconnection from God), financial burden (heavy costs of care, lack of insurance support), and declining social status (negative attitude of the society, suffering from discrimination and inequality).

**Conclusion:**

The consequences of PTSD-related injuries in veterans directly and indirectly affect the overall living conditions of their spouses. These spouses experience emotional detachment and constant rejection, leading to a decrease in their resilience against existing stressors and exposing them to disruptive and challenging issues in individual, family, and social dimensions of life that affect their physical and mental well-being. Therefore, these spouses require empowerment and access to social support in dimensions of educational, caregiving, therapeutic, and supportive. It is recommended that health policymakers pay special attention to designing up-to-date interventions to enhance the health of these spouses in physical, mental, spiritual, and social dimensions.

## Introduction

Post-traumatic stress disorder (PTSD) is the occurrence of specific symptoms after experiencing or witnessing one or more very severe or unfortunate stressful events with the possibility of real death or the threat of death and severe injury [1]. It is a chronic mental disorder that may cause a person to feel fear, disorder or panic after seeing or experiencing a traumatic event [2, 3]. According to the DSM-5, people may encounter such stressful events in one of the following ways: experiencing the event directly, witnessing the event for others, learning that an unfortunate event has happened to a loved one or close friend. War, physical attack, terrorist attacks, and natural disasters are all examples of traumatic events [2]. four specific symptoms of this disorder are: intrusions, avoidance, negative alterations in mood and cognition, and alterations in arousal or reactivity. These symptoms must persist for more than 1 month and cause functional impairment to be diagnosed with PTSD [2, 3]. PTSD is the most common mental disorder among veterans returning from war [4]. This disorder occurs mainly in the first two years after experiencing trauma [5] in 9–25% of war casualties [6], and its prevalence in Vietnamese soldiers is 30%, in women [7] and men who served in Iraq [8], 14%, and the prevalence of this disorder has been reported as 14.9% in the Iranian military personnel [9]. According to available medical records, more than 80% of Iranian veterans have been diagnosed with PTSD [10].The most mental problems are reported among people who were often present on the front lines of war [11].

According to conducted studies, this disorder can have unpredictable, destructive, and irreparable consequences and outcomes for veterans, their family members, and the community [12, 13]. These, in turn, result in significant indirect costs in terms of physical, mental, and social damages for both veterans and their family members, especially their spouses [14, 15]. The spouses of these veteran’s act as like a refuge against these issues and are actually a way for the family to connect with the society [16, 17].

Evidence shows that PTSD veterans often have problems such as anger, aggression and lack of intimacy between spouses. Lifelong contact with these veterans, having the roles of caregiver, wife, mother at the same time attending to the needs of children, taking care of other family members, being in charge of all the duties and responsibilities of life alone, they face problems in their daily life that lead to these people not having a normal routine in life like other members of the society. As a result, these individuals, unlike other members of the society, do not have a typical routine in their lives [18–20]. In fact, these spouses have a unique position because, despite having conditions similar to other individuals in society, they are particularly involved in dealing with the consequences of physical and mental disabilities endured by the veteran. This, in turn, threatens the well-being of these spouses and results in a decrease in their quality of life [19, 21]. The results of a study conducted by Ghahramani et al. in Iran in 2022 revealed that spouses of veterans experience a low quality of life, particularly in terms of their mental well-being compared to other dimensions. Thus, it is crucial to pay attention to the families of veterans, especially their spouses [22]. Similarly, the findings of a study by Mousavi and colleagues in 2021 in Iran indicate that spouses of veterans with psychiatric disorders and PTSD obtained significantly lower scores on various subscales of the quality of life [19, 23, 24]. Moreover, a study by Allen et al. in Croatia in 2018 demonstrated the detrimental impact of chronic PTSD in veterans on the physical and mental health, social relationships, and overall quality of life of their spouses [25].

The spouses of veterans with PTSD, as indirect victims of war and the closest individuals to these patients, play a vital and key role in the lives of these veterans [26]. They face numerous unavoidable challenges in their social, professional, and family lives, and neglecting these challenges and problems can have detrimental effects on their well-being and functioning [18].

One of the important factors that play a role in the interactions of family members is family dynamics. Family dynamics are defined as unique methods used by each family member to interact within the family [27].

This theory holds that family interactions are interconnected like the links of a chain, it is not possible for one family member to experience difficulties without affecting other family members [28]. Furthermore, the family is the primary source of emotional support during psychological and social pressures. When this source encounters problems, each family member becomes exposed to considerable psychological and social pressures. Therefore, these pressures can have a negative impact on the function of family members and cause a crisis in the family [29].

Since the mental effects caused by war are chronic and progressive and continuously affect the quality of life of the affected person, family members, especially their spouses who try to support the veteran [30, 31].

Previous research indicates that the challenges faced by spouses of veterans with PTSD have not been directly studied as a distinct topic until now. Considering that these spouses are among the most influential individuals in promoting the health and improving the quality of life of family members, and considering that most studies conducted in Iran have primarily focused on veterans and fewer studies have specifically targeted the spouses who are often the forgotten victims of war, it is essential to address their specific challenges [32, 33]. Wives are an integral part of veterans’ lives, unfortunately, as a result of living with the injured person for a long time, signs and symptoms of post-traumatic stress disorder are observed in them [34]. Also, considering the cultural and religious differences of the Iranian society, especially in this group of people, and the existence of a gap between theoretical and practical knowledge among the life experiences of the veterans’ caregivers, these experiences can be obtained with the help of a qualitative study [35]. This study was conducted to answer the question, “What are the challenges of living with a veteran diagnosed with PTSD and what effect does it have on their personal, family and social life? " In other words, the research team intended to elucidate the challenges faced by spouses of veterans with PTSD in order to design targeted interventions to improve their mental well-being. Therefore, it is necessary to deeply explore these challenges through a qualitative study. Qualitative research data are of a subjective nature, derived from the perceptions and beliefs of the study participants. As a result, these studies provide a valuable approach to describe lived experiences and contribute to understanding human experiences [36, 37]. By explaining the challenges faced by the wives of PTSD veterans, a more comprehensive view and knowledge can be obtained on the problems of this group of women, who have received less attention, also the identification of these challenges can be the basis for further studies for effective planning to improve the performance of these women, because these women are close The most important people are considered to be veterans, and by improving the performance of these women. Regarding the above mentioned issues, we designed this qualitative study with the aim of exploring the challenges of living with a veteran diagnosed with PTSD from the perspective of their spouses.

## Materials and methods

### Type of study

A qualitative study with a conventional content analysis approach was conducted from June 2022 to January 2023. In this method, information is directly obtained from the participants without preconceived ideas, imposed opinions, or predetermined theoretical perspectives. The knowledge generated through this approach is based on the perspectives of the participants and obtained from the data derived from the interview transcripts [38].

### Participants and research setting

The research population consisted of the spouses of veterans with PTSD. The inclusion criteria for participants in this study were women whose spouses were veterans diagnosed with PTSD by a psychiatrist, who were in a current marital relationship at the time of the study, were able to communicate in Persian, had no hearing problems, and had at least basic literacy skills in reading and writing. The exclusion criteria were severe mental disorders and receiving psychiatric treatments, an obvious physical disorder that leads to the creation of limitations and challenges in the individual, so that due to this disorder, the veteran does not have the necessary ability to seek care, for example, paralysis., and withdrawal from the study at any time of the study. In this study, 15 spouses of veterans who spoke Farsi were selected. The research was conducted at the Veterans Affairs Center in Iran. This center serves as the main source of information and services for veterans returning from war in Saveh County. Due to easy access to the research population and the participants being covered by this center, it was selected as the research setting.

The process of selecting the participants was such that the researcher was present at the Department of Veterans and after obtaining permission from the head of the center to study the files of the veterans, she first identified the veterans suffering from post-traumatic stress disorder and then made a phone call to their spouses, and receiving information from them, if they met the entry criteria and were willing to participate in the study, while explaining the objectives of the research, he invited them to an interview to get their experiences. The average duration of each interview ranged from 30 to 45 min Three interviewees were re-interviewed.

### Data collection method

In order to collect the data, semi-structured, in-depth, face-to-face interviews were conducted in a quiet room without any environmental distractions at the Veterans Affairs Center. The interviews commenced with open-ended questions and were purposefully guided to explore the challenges of living with a veteran diagnosed with PTSD. The interview method was flexible, allowing the researcher to gather more detailed information compared to other methods. The interviews were conducted based on an interview guide developed by the research team, which included questions such as: “please describe your experiences of living with your veteran’s husband” and “please explain your difficulties and challenges in your life” and “How has your husband’s condition affected your family life? ”and “How has living with this person affected you socially?” Probing questions were also used during the interviews to ensure the researcher’s understanding of the participants’ statements, encourage further elaboration, and explore emerging topics. Unlike the main study questions, probing questions were not predetermined [39]. Probing questions were asked for further clarification (e.g. “What do you mean?”, “Will you elaborate further?”). Silent probes allowed participants to reflect on descriptions Towards the end of each interview, participants were invited to share any additional thoughts, and the possibility of future interviews with them was discussed. All the interviews were conducted in Persian by the first author, a doctoral student trained in healthcare qualitative research. Data collection and analysis were simultaneous and during this process the topic guide was modified to explore emerging areas of interest. The participants were also asked for their permission to be contacted if the researcher had any further questions. Data collection continued until data saturation was reached, meaning that in the last two interviews, participants reiterated ideas similar to the information obtained from previous interviews, and no new concepts or categories emerged. Data saturation occurs when no new information can be extracted from the data, and the categories have reached their maximum possible development [40]. The participants’ interviews were recorded using an audio recording device with their full awareness and consent. Subsequently, the participants’ conversations were transcribed verbatim as soon as possible after each interview.

### Data analysis

The data were analyzed using the conventional content analysis approach. Content analysis is a qualitative data analysis method used to categorize sentences. It is a research method to describe the content derived from communication systematically and objectively [41]. In this study, data analysis was performed using the Graneheim and Lundman’s approach, which involves five steps for analyzing qualitative data: (1) transcribing the interview completely immediately after each interview, (2) multiple readings of the entire transcripts to gain a general understanding of the content, (3) Identifying meaning units and basic codes, (4) categorizing initial codes into broader categories, (5) determining the main theme of the categories [42]. To understand and immerse in the data, the transcripts were read multiple times to develop a comprehensive understanding. Each section of the text was then assigned a name or code, and notes were made in the margin. The codes were then compared for similarities and differences, and codes that were similar or had similar meanings and concepts were grouped together. Based on this process, initial categories of codes were developed, and all categories were compared and classified. The classification process continued, and in the end, subcategories and main categories were derived. The coding and categorization process of categories and subcategories was reviewed and revised in multiple research group sessions. This process continued until the group reached agreement on coding and categories, and ultimately, by repeated examination of the categories and contemplation of the overall concept derived from each of them, the underlying meaning of the text and the main category were extracted. we used MAXQDA 2020 for managing codes, themes and memos. Memos were written throughout the analysis process to capture thoughts and reflections of the spouses of veterans with PTSD statements.

### Trustworthiness of data

In this study, the four criteria of Lincoln and Guba including credibility, transferability, confirmability, and dependability were used to check the validity of the data [43]. To ensure the credibility of the findings, we used debriefing by peer and member check methods. In the case of peer debriefing, we had a request to peers who were not researchers on the study but had critique expertise in the field of Veterans PTSD including Psychiatrist and psychiatric nurse. The research team committed themselves to adhere to the topics of the peers and take their opinions into account in the final conclusions. In order to member check, a summary of the interview was returned to the participants to confirm or refute the accuracy of the researcher’s perception. In cases of disagreement between the perception of researchers and the opinion of the participant, we tried to reach a common understanding about the subject through interaction with participants. To improve the transferability of the findings, purposeful and heterogeneous sampling was used in terms of Age, education, Employment status, Income. Audit trial and peer review were used to control the dependability of data. Our research team with different expertise reviewed, revised, and confirmed all encoded data and determined subcategories and categories to confirm the confirmability of the data.

Initially, the study objectives were explained to the participants, and written informed consent was obtained from those who were willing to participate in the study. Additionally, permission was obtained to record the interviews using an audio recording device. Furthermore, the participants were assured of the principles of confidentiality, anonymity, voluntary participation in the study, and the freedom to withdraw at any time without any consequences. The participants were actively involved in determining the time and location of the interviews, ensuring a collaborative approach.

## Results

Based on the findings, the mean age of the participants in the study was 56.72 ± 6.34 years. Furthermore, 33.33% of the individuals had a primary education, 60.00% were housewives, and 66.66% had 1 to 3 children. Additionally, 53.33% of the participants expressed that their income was not sufficient for a decent life. More details are presented in Table 1.

**Table 1:** Participants’ characteristics

Characteristics		N (%)
Age (Years)	40–50	(20.00 )3
	50–60	(53.33)8
	70–80	(20.00)3
	Total	(100)15
Educational level	Primary	(33.33 )5
	Middle	(33.33 )5
	diploma	(20.00)3
	University	(13.33)2
	Total	(100)15
Employment status	Housewife	(60.00)9
	Employed	(13.33)2
	freelance job	(26.66)4
	Total	(100)15
Income	enough	(20.00)3
	enough to some extent	(26.66)4
	not enough	(53.33)8
	Total	(100)15
Number of children	no children	(13.33)2
	1-3children	(66.66)10
	More than 3 children	(20.00)3
	Total	15(100)

The analysis of the interviews led to the extraction of 721 initial codes, which were further reduced to 172 codes through repeated reviews of the codes and comparison of their differences and similarities. Finally, these codes were categorized into sixteen sub-categories and seven main categories related to the spouses’ perspectives on the challenges of living with a veteran diagnosed with PTSD. These categories included burnout, apathy towards self-care and caring for the veteran, depression, Crushed and ignored, relationship disturbances, financial burden, and declining social status, as shown in Table 2.

**Table 2:** Spouses’ perspectives on the challenges of living with a veteran suffering from post-traumatic stress disorder (PTSD)

Main category	Subcategory	Code	Number of code
Burnout	Sleep disturbances	- Low-quality sleep - Difficulty falling asleep - Chronic insomnia - A sense of insecurity	2 2 2 3
	Feelings of exhaustion	- Continuous fatigue - Boredom - Difficulty performing household - A sense of - Lack of strength and energy - Worsening physical problems	3 2 3 3 2 2
Apathy towards self-care and caring for the veteran	Neglecting self-care	- Ignoring physical problems - Disregarding personal mental well-being - Lack of self-care - Lack of motivation for self-care - Neglecting oneself	4 4 3 3 2
	Lack of interest in continuing care	- Feeling annoyed - Lack of appreciation - A sense of indifference towards continuing caring for veteran - Leaving the house	3 2 3 1
Depression	Feelings of hopelessness and being at the end of the line	- Lack of enjoyment in life - Improper mental state - Suicidal thoughts - Death wishes - Meaninglessness of life - Decreased motivation - Insufficient emotional connection - A sense of being defeated - Lack of purpose	2 3 2 4 3 4 - 2
			3 5 4
	Decreased self-confidence	- Loss of self-confidence - Lack of trust in one’s abilities - Difficulty expressing opinions	3 2 2
Crushed and ignored	Being mistreated	- Being physically assaulted by veteran - Physical insecurity - Fear and anxiety	3 2 3
	Having multiple roles	- Compensating for paternal roles - Shouldering all responsibilities - Managing household tasks alone - Acting as intermediaries	2 4 2 2
	Having a sense of self-sacrifice	- Comprehensive caregiving - Self-sacrifice - Concealing pains - Maintaining appearances - Disregarding personal interests - Sacrificing to preserve the family’s life - Letting go of arguments	3 2 2 3 2 2 2
Relationship disturbances	Dissatisfaction with marital relationship	- Lack of desire for sexual intimacy Decreased quantity and quality of marital relationship	2 2
	Isolation and limited social interactions	- Social withdrawal - Inability to establish effective communication	2 2
	Disconnection from God	- Feelings of being abandoned - Lack of peace - Anger towards God	3 2 2
Financial burden	Heavy costs of care	- High cost of treatment - High counseling fees	4 3
		- Inadequate income	8
	Lack of insurance support	- Insufficient insurance support	2
Declining social status	Negative attitude of the society	- Reduced social status, - Unfair judgments - Negative attitude of the society	3 2 5
	Suffering from discrimination and inequality	- Weakness in the support provided by the available resources in the society - Discrimination and injustice - Limited access to healthcare services	2 3 3

### Burnout

Given that the spouses of veterans are constantly in the role of caregivers and repeatedly face the challenges associated with the veterans, and considering the significant responsibilities they have within the veterans’ families, caring for these individuals can lead to burnout over the years, both physically and mentally.

In this study, the burnout category included two subcategories: sleep disturbances and feelings of exhaustion.

*Sleep Disturbances The spouses of the veterans stated that due to the sleep disorder, which is one of the common problems in the veterans suffering from post-traumatic stress disorder, they also experienced sleep problems such as* low-quality sleep, difficulty falling asleep, chronic insomnia, and a sense of insecurity.

The participants expressed their experiences in this regard, “I can’t sleep due to the intensity of stress. I may sleep for about 4 to 5 hours during the whole day, but I never have a deep sleep within that duration.” (P. 5)

I wake up at the slightest sound during sleep. Sometimes, I wake up multiple times during the night and go back to sleep. When I wake up in the morning, it feels like I haven’t slept all night. (P. 7)

*Feelings of exhaustion* A number of participants stated that they have experienced constant feeling of tiredness, inability to do household chores, lack of strength and energy, aggravation of physical problems caused by the maintenance and care of veterans.

One of the participants expressed it as follows, “I constantly have headaches, back pain, and leg pain all the time. My lower back bones have deteriorated, and I have taken many medications, but I don’t get better. Even though I’m not really old, because I have been doing heavy work since a young age and taking care of my spouse, I have ended up like this. I’m always tired, I have no motivation or energy for anything.” (P. 2)

I feel that in recent years, I have become very incapable. It’s not that I’m being lazy, no, I just don’t have enough energy and strength to get up and do my work. Most of the time, my tasks remain unfinished, and it frustrates me. (P. 13)

### Apathy towards self-care and caring for the veteran

In addition to caring for their husbands, the spouses of veterans with PTSD are also responsible for raising children and attending to all daily life tasks and responsibilities. These responsibilities signify the challenging roles these spouses have in their lives. Over time, these factors can lead to indifference and neglect towards self-care and the care of the veteran.

This category contained two subcategories: neglecting self-care and lack of interest in continuing care.

*Neglecting self-care* The spouses of veterans with PTSD, due to their continuous engagement in caring for their spouse and fulfilling multiple responsibilities, often neglect their own health, both physical and mental. This self-neglect can put these spouses at risk of developing physical and mental illnesses. Ignoring physical problems, disregarding their own mental well-being, lack of self-care, lack of motivation for self-care, and neglecting oneself were other challenges of living with a veteran diagnosed with PTSD. In this study, the participants believed that they lacked sufficient time, motivation, and conviction for self-care.

They expressed their experiences as follows, “…… I went to several doctors, and most of them said I needed surgery. But I wonder, if I undergo surgery, I’ll have to spend some time in bed. Then who will take care of this life and the children? Who will do my tasks? My husband is also unwell so I can’t expect him to help.” (P. 4)

I’ve given up on myself, may God protect me. Just being able to manage the issues and problems of my life is a lot. I don’t have time for myself anymore.” (P. 5)

*Lack of interest in continuing care* Feeling annoyed with the veteran and children, lack of appreciation from the veteran, and experiencing a sense of indifference towards continuing caring for the veteran were among the challenges of living with veterans suffering from PTSD. In this study, some of the participants believed that they had become disinterested in continuing to take care of the veteran, which could be attributed to emotional detachment and the veteran’s lack of appreciation for their efforts and sacrifices.

The participants expressed their experiences as follows, “I try my best to take good care of him, show him love, and give him his medications on time. But who appreciates it? Who knows the value of these hands that are worn out?” (P. 14)

I have always sacrificed so much to take care of him, neglecting my own needs and interests, just to ensure I can take good care of my husband. But it’s a shame that he doesn’t even recognize my love and care. There have been times when, due to his aggressive behavior that threatened my life, I had to leave home, but my heart couldn’t stay away, and I came back.” (P. 9)

### Depression

Since veterans with PTSD themselves face significant psychological and social challenges, it is highly likely that their family members are also at risk of experiencing psychological harm [44]. Among them, the spouse of the veteran is the primary emotional and psychological support for the veteran and is the first person directly exposed to secondary trauma and cognitive difficulties [45] According to the interviews, the spouses of veterans with PTSD have been involved in some psychological problems due to the problems and hardships of their husbands.

This category included two subcategories: feelings of hopelessness and being at the end of the line, decreased self-confidence,

*Feelings of hopelessness (being at the end of the line)* Lack of enjoyment in life, improper mental state, boredom, suicidal thoughts, death wishes, meaninglessness of life, decreased motivation, and insufficient emotional connection were among the challenges experienced by most participants in living with veterans suffering from PTSD.

Also The participants stated that they experienced a lack of hope in life, a sense of being defeated, and a lack of purpose. They argued that due to long-term exposure to the challenges of living with a veteran suffering from PTSD, they had lost their hope.

The participants expressed their experiences in this regard, “I ask my spouse if he mistreats me because he thinks I am his prisoner. Sometimes, I pray to God for death to find peace.” (P. 4)

To be honest, life has no meaning for me anymore. I have no motivation to continue living. Sometimes I ask myself how I’m still alive with all these problems. I should have died a hundred times by now, but…” (P. 15)

The participants shared their experiences in this regard, “I feel like a lifeless being. I just go through the days without any meaning. There is no joy, no purpose, no aspiration. I simply have nothing anymore.” (P. 8)

*Decreased self-confidence* The loss of self-confidence means lack of trust in one’s abilities and difficulty expressing opinions, which were among the challenges of living with a veteran suffering from PTSD, as expressed by some of the spouses in this study.

In this regard, one of the participants stated, “He made me stay at home all the time. I had become so secluded and my self-confidence had deteriorated to the point that I couldn’t even utter a single word outside or in family gatherings.“(P. 1)

### Crushed and ignored

Despite their significant efforts to meet the needs of the veterans with PTSD and their families and juggling multiple roles simultaneously such as being a spouse and a mother, these spouses are unfortunately often overlooked and not given the appreciation they deserve. Not only are their efforts unacknowledged by the family members and the veterans themselves, but also they are mistreated and beaten by the veterans in many cases. This leads to a feeling of being alongside a sense of being overlooked.

In this study, this category had three subcategories: being mistreated, having multiple roles and having a sense of self-sacrifice.

*Being mistreated* Veterans suffering from PTSD are more sensitive than ordinary individuals due to the chronic nature of the illness and their mental engagement with traumatic war memories and psychological challenges. They are quick-tempered people who react very quickly to environmental stimuli and become aggressive [46]. Violent behavior is one of the consequences of veterans being placed in complex situations, which makes family members, especially their spouses, experience physical violence and psychological insecurity [47]. In this study, being mistreated refers to physical assault by the veteran, lack of physical safety, and fear and anxiety, with the spouses of veterans being the primary victims.

In this regard, the participants shared their experiences as follows, “My husband once physically assaulted me over a trivial matter. He hit me so hard that two of my teeth broke. Since then, I’m afraid to argue with him because when he gets angry, he loses control over his behavior.” (P. 12)

*Having multiple roles*, the participants stated that due to their spouse’s problems, they play multiple roles that sometimes conflict with each other.

In this regard, one of the participants said, “I’m exhausted from doing everything myself. Most household responsibilities, such as grocery shopping, clothing, etc. are on my shoulders. Although I make a great effort to manage everything well, sometimes I feel overwhelmed and on the verge of collapse.” (P. 6)

I was both the homemaker and the breadwinner. I had to carry the entire burden of life on my shoulders and handle both household and external tasks alone. Moreover, my spouse required constant care, and I had to take care of many personal matters on his behalf. (P. 4)

*Having a sense of self-sacrifice* One of the forms of emotional support that the spouses of veterans offered to them was self-sacrifice in various ways. They selflessly dedicated themselves physically, emotionally, and mentally to their spouses. However, due to the lack of recognition and understanding of their sacrifices and selflessness, these spouses developed a sense of self-neglect in their own lives. Their comprehensive caregiving, enduring suffering, self-sacrifice, concealing their pains, maintaining appearances, disregarding their own interests, sacrificing to preserve the family’s life, and letting go of the arguments were among the experiences shared by the participants.

In this regard, one of the participants said, “With all his anger and our arguments, I sacrificed myself for this life.” (P. 12)

Many people tell me that I have dedicated myself to my husband and children, that I am a generous and compassionate person who has managed to live all these years in such circumstances. (P. 3)

### Relationship disturbances

Given the hardships that spouses of veterans with PTSD endure and the neglect they face from both family and society, Especially the veteran, they gradually experience disruptions in their intrapersonal and interpersonal relationships(including husband and wife relationships), as well as in their relationship with God [48].

This category had three subcategories, including Dissatisfaction with marital relationship, Isolation and limited social interactions and disconnection from God.

*Dissatisfaction with marital relationship* in a healthy marriage, the presence of a satisfying sexual relationship plays a significant role in maintaining the stability of the family unit. In veterans, sexual relationships and functioning may vary depending on the type and severity of their trauma [49]. Some spouses experienced a lack of desire for sexual intimacy and a decrease in the quantity and quality of their marital relationship, which, in turn, resulted in marital dissatisfaction and a growing distance between the husband and wife.

The Participants in this study expressed their experiences in this regard, “The mental condition of my spouse has also affected our intimate relationship to the point that I have no desire or patience for sexual intercourse.” (P. 14)

My spouse and I haven’t had any sexual relationship for about 5 years or more, and it all goes back to the psychiatric medications that he takes. (P. 8)

*Isolation and limited social interactions* Isolation and relationship disturbances were among the challenges mentioned by the majority of participants. In fact, one of the problems and hardships that spouses of veterans with PTSD endure is a sense of loneliness, which leads to a lack of connections with others.

In this regard, one of the participants said, “My interactions with relatives and friends have become limited. Relatives don’t drop by because of my spouse’s bad behavior, and I and my children are hesitant to visit relatives because he might suddenly cause a scene there.” (P. 6)

For several years, I have lost interest in socializing and connecting with others. This man has taken away all my motivation. (P. 11)

*Disconnection from God* based on the interview findings, the spouses, due to living with a veteran suffering from PTSD for a long time, experience a disconnection from God in various forms. Feelings of being abandoned, lack of peace, and anger towards God were expressed by the participants. This disconnection from God is experienced as a sense of unrest in these individuals.

In this regard, one of the participants said, “Every day I pray and ask God to help this man get on the right path, to bring his senses back so that our life can improve. But when I realized that God didn’t listen to my voice, I became very angry with Him, and I kept asking why He treated me this way, as I haven’t been a bad servant.” (P. 7)”

### Financial burden

The participants stated that the absence or lack of money caused them to experience many financial problems. Veterans suffering from PTSD who are unable to work and have high medical expenses because the families of these veterans experience many financial problems in addition to physical illness due to the veteran’s mental illness. In this study, financial burden was classified into two subcategories, including heavy costs of care and lack of insurance support.

*Heavy costs of care* Many participants complained about the high cost of treatment and counseling fees as well as inadequate income. Veterans suffering from PTSD not only experience the physical and psychological effects of the illness but also suffer from the financial burden of their care. Their families are also concerned about the high medical expenses in addition to the challenges of caring for the veteran.

In this regard, one of the participants said, “Our income is meager, and it all goes for medication and treatment for my spouse. There’s nothing left for us.” (P. 13)

These veterans are in great need of counseling services. For each half-hour session my spouse attended, we had to pay 70,000 tomans. We had to go at least twice a week. I took my spouse for a few sessions, but I couldn’t afford to continue the treatment. My spouse only has a retirement pension, which barely covers our living expenses amidst these high costs. (P. 8)

*Lack of insurance support* Insufficient insurance support was among the issues mentioned by the majority of participants. According to the data, insurance only covers limited services such as occupational therapy, physiotherapy, and physician visits. However, it does not fully cover many services needed by these veterans, including medication costs. The participants stated, this issue has made their life situation worse. *One of the participants said,*

It’s true that my spouse has supplementary insurance, but most of the time, when we take my spouse for treatment, either it’s not covered by the supplementary insurance, or we have to pay out of pocket and then submit the invoices to receive a small percentage of the expenses several months later. It’s very difficult for us in this situation. (P. 13)

### Declining social status

This category refers to how the valor and sacrifices of veterans were initially valued while the values have diminished over time.

This category encompassed two subcategories, namely negative attitude of the society, and suffering from discrimination and inequality.

*Negative attitude of the society* The reduction in the social status, unfair judgments, and negative attitude of the society were among the experiences reported by the majority of participants. They believed that Veterans suffering from PTSD due to many psychological problems and their families are considered as distinct social groups whose social status and respect are not adequately by some individuals. They had experienced negative treatment from some people towards veterans and their families.

In this regard, one of the participants said, “People keep their distance and say, ‘You’re lucky, the government takes care of everything for you. What worries do you have?’ But they don’t know that we are burning from within ourselves, regardless of how others perceive us…” (P. 3)

People don’t know how much we struggle. They think we have no worries or problems in our lives. They say, ‘You went to the frontlines once in your life, so what? It’s nothing now.’ But it lasts a lifetime. They think being a veteran is a ladder to your own and your children’s progress. (P. 1)

*Suffering from discrimination and inequality* The sense of discrimination among Families of veterans suffering from PTSD disorder refers to the feeling of injustice and in receiving privileges related to these people and their families, as well as the lack of proper access to resources and services in the society.

One of the participants said, “Because my spouse’s disability rating is below 25%, we don’t qualify for many benefits and resources, neither for us nor for our children. We don’t even have the right to object. It sometimes feels like we are being insulted.” (P. 14)

I strongly disagree with these percentage-based classifications. I know people whose situations are much better than my spouse’s, but they managed to get a higher rating through favoritism. It’s okay. I always tell my spouse that you went to war for the sake of God, entrust everything to Him. (P. 9)

## Discussion

The purpose of this study was to explore the challenges of living with a veteran suffering from PTSD from the perspective of their spouses. The identified challenges were categorized into seven categories, burnout, apathy towards self-care and caring for the veteran, depression, Crushed and ignored, relationship disturbances, financial burden, and declining social status.

After the war and the return of veterans to their families, the stressors arising from the war and their negative impact on the physical and mental well-being of the veterans and the family members, especially the spouses who repeatedly experience the turmoil of the afflicted person, persist [50]. These experiences lead to a reduction in the quality of life of the family members, particularly the spouses of these veterans [51]. The family acts like a system because the behavior of the members of a family is a function of the behavior of other family members, and if there is a problem in the behavior and states of one of the members, the balance of the family is disrupted. The influence of one member of the family on another member is obvious and clear, so that it is not possible for a person to have a problem in a family and this problem does not affect other family members [52]. Therefore, the families of PTSD patients are in a more difficult and unstable situation emotionally, which leads to burnout in the functioning of the family and presents challenges and crises over time [4]. These challenges were clearly observed in the findings of the present study, which were based on the interviews with participants (spouses of veterans with PTSD).

Sleep disturbance is one of the primary and most common complaints among spouses of veterans PTSD. Because veterans have behaviors such as nightmares, frequent waking up, severe insomnia, etc., they cause suffering and discomfort to their spouses and as a result, sleep disorders in them [53]. According to the findings of a study by Hojjati et al. in Iran (2017),the lived experiences of the spouses of veterans indicate a decrease in their sleep quality. These spouses, due to marital commitment, are concerned about the condition of their spouse even during sleep and unconsciously wake up at night to be present beside them [51]. These findings are consistent with the results of the present study, as the participants expressed that due to the unique and specific circumstances of their spouses, they were lacking sufficient, suitable, and quality sleep for a long time and suffered from chronic insomnia. This sleep disturbance, in turn, exacerbates the challenges they face in various aspects of their lives, including physical dimensions.

Nursing and caring for disabled people such as veterans, especially veterans suffering from post-traumatic stress disorder, causes the caregiver, who in most cases is the spouse of a veteran, to suffer from many physical problems. Some of these physical problems have psychological roots and others are caused directly by the patient [54]. The results of the present study revealed that the Conditions of veterans with PTSD veterans with PTSD had an impact on the physical and psychological well-being of family members, especially their spouses, who have continuous and long-term contact with these stressors, leading to feelings of fatigue, inability to perform household tasks, lack of capacity and energy to engage in activities, and consequently, exacerbation of physical problems. Polenick et al.in United States (2017) showed that living with physically and mentally disabled people causes physical problems in the spouse [55] and Akhoondzadeh et al.in Iran (2017) showed that the spouses of veterans with PTSD experienced significant physical, emotional, and psychological challenges due to their continuous and repeated interaction with the veterans [56]. These results are in line with the results of the present study.

Furthermore, Braš et al.in Croatia (2019) found that the spouses of veterans were significantly affected by the physical disabilities and psychological consequences experienced by the veterans [57]. These challenges, over the long term, disrupt family functioning and give rise to common issues such as shame, guilt, mistrust, and reduced intimacy within the family [19]. The enduring nature of these challenges can not only give rise to physical problems but also have an impact on the mental well-being of spouses, resulting in distress and the development of psychological disorders [58] The results of a study by Owens-King et al. (2019) revealed that the spouses of these veterans can be considered among the at-risk groups in terms of mental health [59]. The highest prevalence of psychological problems among these spouses is related to depression and anxiety [60]. Additionally, the findings of a study by Heydari et al.in Iran (2022) showed that the spouses of veterans with PTSD experienced more psychological consequences of post-traumatic events compared to other family members, since the hyperarousal symptoms of veterans contribute to serious impairments, such as stress and depression, in their spouses, leading to high levels of distress [61]. These findings are consistent with the results of the present study, highlighting that these spouses experience challenges such as hopelessness, reduced self-confidence, and a sense of sacrifice, which they struggle to cope with.

Beks et al.in Canada (2018) found that the level of violence and aggression, particularly domestic violence towards spouses, was significantly higher in families of veterans with psychological injuries compared to other families, which could be attributed to the heightened irritability of the veterans [62]. These findings are in line with the results of the present study, where the spouses expressed that despite their significant sacrifices in caring for the veterans, accepting additional caregiving roles alongside other heavy responsibilities, financial concerns, reduced social support, adaptation to adverse life circumstances, they experienced repeated physical abuse and violence from the veterans. They added that these experiences not only created feelings of insecurity, fear, and anxiety but also contributed to a sense of being sacrificed in life and ultimately led to disinterest in self-care and caring for the veteran. The persistence of these issues and challenges results in a sense of neglect and feeling overwhelmed, leading to impaired functioning of these spouses within the family. These findings are consistent with a study by Borjali et al. in Iran (2021) in this regard [63].

One of the challenges expressed by these spouses was that the veteran’s condition had affected intimate relationships within the extended family. These spouses must constantly play the role of a mediator between the veteran and the children and always maintain a support role for both parties, which creates additional pressure. Moreover, these veterans exhibit behaviors such as lack of interest and motivation in socializing with others, and they also face difficulties in their communication with others. As a result, the spouses of these veteran’s experience limitations in their interactions with others and subsequently become isolated. These findings are consistent with the results of a study by Khodabakhshi et al. in Iran (2019), which indicate that the pressures arising from providing psychological and physical care to the veteran also have negative effects on the relationship between the mother or spouse as the primary caregiver and the children and acquaintances [64].

Post-traumatic stress disorder may cause a veteran to be unable to perform his marital duties and tasks, and on the other hand, due to inefficient behaviors, may create conditions that cause problems in married life [65]. Allen et al. in United States (2018) found that the spouses of elderly veterans with PTSD had lower marital intimacy compared to other couples [25],which is consistent with the findings of the present study. The majority of participants expressed dissatisfaction with their marital relationship, which led to reduced life satisfaction, loss of hope in life, and decreased individual, psychological, and social functioning of the spouses over time. However, it should be noted that not all participants mentioned this issue; they neither explicitly reported a lack of satisfaction with their marital relationship, nor did they highlight a decrease in emotional and psychological intimacy between them and their veteran spouses.

The majority of the participants stated that when they faced challenges in life, they found solace in their spiritual and religious beliefs, which in turn created motivation and hope for them. They believed that the spiritual rewards of such practices were significant to them, rather than worldly rewards. As a result, they never surrendered to difficulties. However, some others expressed that sometimes the problems and issues were greater than what could be solely addressed by these religious beliefs. Consequently, it can be argued that individuals may exhibit different reactions. Some may resort to spirituality and seek help from God, accepting these challenges as divine tests, while others may reduce or sever their connection with God. These findings were evident in the results of the present study and are consistent with the findings of a study by Yahyazadeh et al. in Iran (2016) [18].

One of the challenges found in the present study was the financial burden, which is in line with the results of the study by Mitter et al. (2017), which showed that living with a disabled adult imposes additional costs on the family [66]. Evans et al. (2019) concluded that homelessness among combat soldiers and veterans has increased and needs a plan to improve [67]. Although veteran’s affairs covers some of the expenses of these people, many veterans do not use these privileges and medical expenses Excess creates problems for them. The results of the present study showed that one of the bothersome challenges for the veterans’ spouses was that the support available in the society for veterans and their families, in addition to being inadequate, also created an inappropriate psychological atmosphere and even led to social exclusion for these families. The attitude of the society and even some governmental institutions towards the phenomenon of veteran and being subjected to unjust judgments constantly offended these families. This, in turn, has caused suffering for the veterans and their children. These findings are consistent with the results of a study by Shafiei et al.in Iran (2022), which indicated that the general public’s stereotypical view towards the financial benefits and social privileges of veterans was often accompanied by bias due to their lack of awareness of the deeper realities. Sometimes, these issues become so intense that these families prefer not to mention their “father being a veteran” because they are faced with unrealistic and unjust judgments from others [68].

The overall belief was that the social status of these veterans has declined, which is consistent with the findings of a study by Khalili et al. in Iran (2018), who stated that the literature on veterans has undergone changes, and in many cases, the sanctity and social status of being a veteran have been diminished to a set of compliments as well as advertising and media discourse [69]. According to the findings of Khodabakhshi et al.in Iran (2019), there have been transformations in the values and perceptions associated with being a veteran and self-sacrifice, leading to a materialistic and livelihood-oriented approach to the extent that, in the overall societal perspective, the material and economic outlook has prevailed over the cultural outlook [64]. This is in line with the findings of the present study and reflects the complaints of the spouses, indicating that despite the limited and insufficient benefits and allocations considered for veterans and the heavy costs of their care, the negative attitudes of individuals in the society persist, leading to a sense of discrimination, humiliation, and deprivation of respect.

Furthermore, feelings of alienation and lack of organizational belonging towards service-providing institutions have led to a sense of deprivation of organizational identity. This dissatisfaction with service-providing institutions is consistent with the results of a study by Shariati et al.in Iran [70], indicating that veterans have gone to war and accepted its consequences due to their special values and beliefs. In other words, the value of self-sacrifice accompanies the insignificance of material rewards for them. This transformation has also added to the cultural concerns and the scope of separation and social isolation among veterans and some family members, especially their spouses and children [71]. Overall, the above factors indicate that the psychological and physical condition of veterans with PTSD casts a shadow over their entire lives, particularly their family life, has led to dissatisfaction among family members, especially the spouses of these veterans, and has created deep wounds in the economic, social, and cultural dimensions of their lives.

### Limitations

The present study, similar to other studies, has limitations. One of the limitations was the trust of the participants, the spouses of the veterans did not have enough trust to express their challenges at first. The researcher tried to control this limitation by interacting with the spouses for a long time and gaining their trust so that they can express their behavior freely. Furthermore, another limitation of this study due to the nature of qualitative studies is that the generalization of the results cannot be considered with certainty.

## Conclusion

The results of the present study demonstrated that the consequences of PTSD-related injuries in veterans directly and indirectly affect the overall living conditions of their spouses. These spouses experience emotional detachment and constant rejection, leading to a decrease in their resilience against existing stressors and exposing them to disruptive and challenging issues in physical, psychological, and social dimensions of life. The spouses of these veterans are at the center of the family and serve as a refuge in the face of life difficulties. In addition to fulfilling their spousal duties, they shoulder the responsibilities of motherhood and other roles, often prioritizing the needs of others over their own physical and mental well-being, in order to regulate the relationships within the family and with the community. Therefore, the identification of these life challenges is considered essential because it enables the implementation of beneficial and constructive measures to empower them in psychological, emotional, and physical aspects. It also allows them to benefit from social support in educational, caregiving, therapeutic, and supportive dimensions, such as counseling and providing appropriate information.

Since the spouses of veterans with PTSD have an important role in maintaining and maintaining the cohesion of the family environment, and with their flexibility and adaptability, they can play an important role in finding family balance and reducing stress and tensions in life. Therefore, support systems such as counseling and providing appropriate information can improve the quality of life of veterans’ spouses and subsequently improve the quality of life of veterans. In the meantime, teaching life skills and communication skills and stress management methods and increasing understanding and social support can be helpful ultimately leading to positive impacts on the society. it is essential to develop precise plans to address and facilitate the challenges faced by these spouses and to empower family members, especially the spouse directly involved in dealing with the difficulties arising from the veteran’s injury. Additionally, it creates the necessary environment and motivation for further research in this field for researchers.

## Data Availability

The datasets used and analyzed during the current study available from the corresponding author on reasonable request.
